# Supplementary material for: Are carbon emissions trading and green financial instruments synergistic? -Comprehensive quantitative research based on content analysis
Source: PLoS One. 2024 Mar 7;19(3):e0298601. doi: 10.1371/journal.pone.0298601 (PMC10919601; doi:10.1371/journal.pone.0298601)
Supplement: S1 Data — (ZIP) [file pone.0298601.s001.zip › Data/地级市Z_F工作报告.zip!/╔╜╢1⁄2/╢1⁄2╙¬╩╨/└┤╫╘╣1⁄2╓┌║┼í╛┬φ┐╦╩2╛▌═°í┐_2005.docx]

各位代表:

现在,我代表市人民政府向大会作政府工作报告,请予审议,并请各位政协委员和其他列席人员提出意见。

　　一、2004年政府工作回顾

　　2004年是不平常的一年。一年来,面对宏观形势发生的重大变化，全市上下在省委、省政府和市委的正确领导下，在历届市领导班子打下的良好基础上，以邓小平理论和“三个代表”重要思想为指导，认真贯彻党的十六大和十六届三中、四中全会精神,牢固树立和落实科学发展观,解放思想，干事创业，在宏观调控中抢抓机遇，在克服困难中加快发展，胜利完成了市五届人大二次会议确定的各项任务，在全面建设小康社会的进程中迈出了坚实步伐。

　　(一)国民经济保持良好发展势头。初步统计,2004年全市实现生产总值891.9亿元,比上年增长17.2%,其中地方423亿元,增长25%。农业和农村经济全面发展，农业增加值增长8.5%，畜牧、水产、蔬菜、冬枣、粮棉等优势产业不断壮大,产业化、标准化水平有了新的提高。防治高致病性禽流感取得阶段性胜利。石油工业稳步发展，地方工业快速增长，全市规模以上工业总产值和销售收入突破千亿元；规模以上工业增加值增长29.5%,其中地方增长55.1%;地方规模以上工业实现利税、利润分别增长56.8%、59.4%。高新技术产业产值占全市规模以上工业总产值的比重提高2.6个百分点。投资力度加大，全社会固定资产投资增长33.1%,其中地方增长53.8%。消费稳中趋旺，全市社会消费品零售总额增长16.4%。民营经济活力增强,个体工商户达到4.7万户、私营企业8914家,上缴税金增长33.8%。财政金融平稳运行,境内财政总收入187.3亿元,其中地方财政收入28.3亿元,按可比口径增长27.5%；各项存款余额555.3亿元,贷款余额357.4亿元,分别比年初增加59.5亿元、62.3亿元。

　　(二)改革开放迈出新步伐。招商引资扎实推进,到位外来固定资产投资73.2亿元。对外经贸进一步扩大，完成进出口总额10.6亿美元,增长26.4%,其中出口6.1亿美元,增长36.5%；实际利用外资40396万美元，其中吸收外商直接投资39896万美元，增长51.5%。成功举办了2004东营经贸洽谈会。东营经济开发区等各类园区加快发展，带动作用明显增强。企业改革不断深化。科达股份成功上市。加大了对油田存续企业改制的支持力度，有21家企业完成改制。农村税费改革深入推进，全部取消了农业特产税,农业税税率降低3个百分点，减轻农民负担6865万元。直接补贴种粮农民737.3万元。投资、财税、价格、土地管理、粮食流通、户籍和农村信用社等方面的改革不断深入，事业单位改革稳步推进。整顿和规范市场经济秩序工作扎实开展,食品、药品、农资、土地市场秩序和开发区、固定资产投资项目等专项治理整顿取得明显成效。

　　(三)城市和基础设施建设进一步加强。城市规划力度加大,编制专业规划8项，完成了一批重点地段、重点区域的详细规划和城市设计。城市建设改造步伐加快，绿化美化亮化和环境综合整治取得较大成效，获山东省适宜人居环境奖。中心城外环路全线贯通。城市管理综合执法工作进一步加强,管理水平有了新的提高。县城和小城镇呈现出良好发展态势，村庄规划和改造取得新的进展。围绕构建交通、能源、防灾减灾等体系,实施了一批重大基础设施建设项目。

　　(四)统筹发展取得明显成效。统筹城乡发展，加大对“三农”的支持力度，农村生产生活条件进一步改善。去年市财政支持基层和“三农”的资金达4.6亿元。统筹经济社会发展，促进了社会全面进步。科教兴市战略深入实施，科技创新能力增强。基础教育、职业教育、成人教育全面发展,教育质量有了新的提高。创建山东省公共卫生安全示范区取得初步成效，疾病预防控制和医疗救治能力进一步增强。文化体育、新闻出版、广播电视事业健康发展，老龄、残疾人保障和妇女儿童工作受到全社会的重视，双拥共建、国防教育、外事侨务、民族宗教、人民防空、无线电管理、气象、地震、史志、档案等各项事业都取得了新成绩。思想道德建设不断深入，群众性精神文明创建活动广泛开展，创建“文明城市”、“小康文明村”工作和“信用东营”建设扎实有效。普法教育和依法治市深入推进。基层民主不断扩大，村委会换届选举基本完成。加强“平安东营”建设，依法严厉打击各种犯罪行为，妥善化解各类社会矛盾,毫不放松地抓好安全生产,保持了社会稳定。统筹区域发展,油地军校结合不断深入。油田工业园已确定入园项目23个。油田帮扶乡镇工作取得新成效。落实各项政策措施,促进了县域经济发展。统筹人与自然和谐发展,人口、资源、环境工作得到加强。人口自然增长率控制在5.25‰。耕地得到严格保护,土地等资源利用效率提高。污染防治力度加大。林木覆盖率提高4个百分点。

　　(五)“双十”工程任务顺利完成。在为民办十件实事方面，通过实施“一池三改”，1万农户用上了安全洁净的沼气；基本构筑起科技下乡服务体系和农村现代远程教育网，培训农民26.2万人；农村“五保”老人集中供养率达到95%，生活补贴由200元提高到400元，并建立了全部由财政负担的供养资金筹措机制；粮食储备能力进一步扩大；基本建立起疾病预防控制和医疗救治体系；为农村中小学配备了一批教学设备,扩建普通高中校舍12.8万平方米，建设改造乡镇中心幼儿园1.9万平方米，救助考上大学的贫困家庭学生542人；改造城乡首集市场14处,建成了农产品市场肉菜粮检测体系；建设了一批治安管理设施，改善了治安管理和案件侦破手段；开通了12319便民服务热线；人力资源市场开工建设。在实施十大工程方面，东营港扩建工程已开工建设;百公里灌区节水改造、百公里骨干河道排水改碱、百公里水域绿化工程全部完成;开发荒碱地35.2万亩；农村绿化工程完成造林面积31.5万亩;建成了政务信息资源管理、空间数据库和异地容灾系统;建设改造城市主次干道90.2公里、干线公路128公里、农村公路418公里，硬化村内主街道212公里,实施了一批市政设施配套工程,建成了市高新区孵化大厦和中试车间,编制完成了300个村庄规划,创建全国无障碍设施示范城通过了省级验收，汽车总站开工建设；快餐店、海鲜城等服务业设施和黄河三角洲国家地质公园规划建设进展顺利;农村有线电视通村率和入户率分别达到97.4%、62.6%；黄河南防潮堤建设完成年度计划;黄河河口物理模型已开工建设。

　　(六)人民群众生活得到较大改善。城镇居民可支配收入达到12935元,增长26.8%;农民人均纯收入4033元,增长14.9%。城乡居民人均储蓄余额18356元,增加2096元。新增就业3.7万人,其中再就业1.3万人,城镇登记失业率控制在2%。初步建立起以城乡养老保险、城乡低保、城乡医疗保险、农村“五保”老人集中供养和教育救助、残疾人救助、灾害救助、住房救助为主要内容的社会保障救助体系，保障能力和水平进一步提高。认真解决征地、拆迁、改制中损害群众利益和拖欠克扣农民工工资等问题,维护了群众的合法利益。

　　(七)政府自身建设深入推进。深入开展“政府效率年”活动,加快转变政府职能，大力加强机关规范化建设，认真落实政务公开、服务承诺等制度，提高了工作效率和水平。全面推进依法行政，促进了法治政府建设。认真贯彻实施行政许可法,对行政许可规定和主体等进行了全面清理，精简审批项目，规范审批行为，提高了审批服务水平。加强督查考核和行政效能监察,开展社会评议市政府部门、单位工作,促进了作风转变和工作落实。加强廉政建设，加大从源头上预防和治理腐败力度，进一步规范了行政行为。

　　去年在矛盾较多、困难较大的情况下，我市经济社会发展创出了建市以来的八个历史之最：全市经济总量增加最多，经济发展的质量最好，固定资产投资规模最大，境内财政总收入和地方财政收入增加最多，城镇居民可支配收入增长最快，农民人均纯收入增加最多，为民办的实事好事最多，安全生产和社会稳定状况最好。在工作中，我们突出把握了以下几点：一是坚持紧紧扭住发展不放松，千方百计加大投入，加快膨胀经济总量。去年固定资产投资拉动全市经济增长10.4个百分点。二是坚持调整优化经济结构，大力推进工业强市，一手抓招商引资，一手抓现有企业膨胀，壮大工业综合实力；大力推进“三荒”开发，以增量促农业结构调整；大力推进服务业设施建设，加快发展第三产业。三是坚持一心为民，把群众的需求作为政府工作的重点，努力为群众解决生产生活中的难事。四是坚持维护社会公平公正和稳定，针对城乡差距、贫富差距较大的实际，不断加大对“三农”、弱势群体和困难群众的倾斜扶持力度，完善社会保障救助体系。五是坚持严格依法行政，改进执法方式，努力解决以权谋私、执法扰民、侵犯群众合法权益等问题，提高依法管理经济和社会事务的水平。这些做法，我们要继续坚持，并在实践中不断完善。

　　各位代表，去年是实施“十五”计划的第四年。到去年我市“十五”计划确定的主要预期目标已提前完成。与“九五”末的2000年相比，地方生产总值、规模以上工业增加值翻了一番多，吸收外商直接投资、外贸出口、地方固定资产投资翻了两番多，地方财政收入、城乡居民储蓄余额接近翻番；地方经济在全市经济中的比重由37.2%提高到47.4%。这四年是我市经济持续高速增长、综合实力实现大跨越的四年，是城乡面貌日新月异、发生巨大变化的四年，是各项事业蓬勃发展、社会全面进步的四年，是人民群众得实惠最多、生活水平显著提高的四年。四年累计实现的生产总值相当于建市以来前十八年的总和，四年再造了一个“东营”。现在，我市经济发展的水平已经进入全省“第一方阵”，列全国百强城市第37位。四年取得了辉煌的成就。这是省委、省政府和市委正确领导的结果,是市人大及其常委会监督支持、人民政协和各民主党派参政议政的结果,是油地军校、全市上下同心同德、艰苦奋斗的结果。在此,我代表市人民政府向全市人民和社会各界,向所有为东营的建设和发展做出贡献的同志们、朋友们，表示衷心的感谢并致以崇高的敬意!

　　在肯定成绩的同时,我们也清醒地看到经济社会发展中还存在一些矛盾和问题,政府工作中也有不少的缺点和不足。主要是农业抗御自然灾害的能力还不强，农民增收的基础还不稳固；地方工业主导产业优势不突出，大企业和名牌产品少；城市功能还不完善，辐射带动能力差；科技创新能力不强，经济外向度不高，影响发展的体制性、机制性障碍仍然存在；城乡差距、贫富差距仍然较大，农村生产生活条件还没得到根本改善，有些群众生活还很困难，有些下岗失业职工还没得到妥善安置，城镇新增劳动力还存在就业难的问题；一些深层次矛盾逐步显现，影响社会稳定的因素仍然较多，社会治安形势不容乐观；有些政府工作人员依法行政意识和能力不强，工作作风不扎实，脱离群众，有的甚至为政不廉、以权谋私。对此，我们一定高度重视，采取切实措施,认真加以解决。

　　二、2005年政府工作的总体要求

　　现在，东营正处在发展的关键时期。这个时期是膨胀总量、提高质量，把全市经济做大做强的关键时期；是培育产业集群，形成明显的产业优势，进而提升整个区域竞争力的关键时期；是提高城市辐射带动能力，实现城乡互动发展的关键时期；是构建和谐社会，实现全面协调可持续发展的关键时期；是在全省率先全面建成小康社会、率先提前基本实现现代化的关键时期。总之，是整合各种资源，发挥各种优势，加快我市经济社会发展的关键时期。今年是这个关键时期的关键一年，我们要在提前完成“十五”计划的基础上推动经济社会发展再上新台阶，要在国家加强和改善宏观调控的形势下继续保持经济快速增长，要制定“十一五”规划、勾画好发展蓝图并为实施这一蓝图奠定坚实基础，做好今年的工作意义重大。综观今年面临的形势，我们既有许多有利条件，也面临一些困难和挑战。国际经济仍将保持较快增长，我国“入世”进入后过渡期，对外开放的步伐将进一步加快，有利于扩大出口和利用外资；国家宏观调控政策和市场机制的完善，将使发展的宏观环境越来越好；我市经济社会发展势头好、后劲足，特别是全市上下发展的热情空前高涨，并探索形成了科学发展的路子，为做好今年的工作奠定了坚实基础。同时国内外竞争日益激烈、国际原油价格走向难以预测、建设用地紧张和资金供应不足等，也将对今年的发展带来一定影响。但总体上看，机遇大于困难，有利条件多于不利条件。我们一定要正确分析和把握形势，坚定信心，抢抓机遇，开拓进取，努力加快发展。

　　今年政府工作的总体要求是:以邓小平理论和“三个代表”重要思想为指导,全面贯彻党的十六大和十六届三中、四中全会精神,坚持以科学发展观统领发展全局,继续加大对“三农”的支持力度，加快壮大工业实力，大力发展服务业，提高城市品位档次和辐射带动能力，着力构建社会主义和谐社会，努力扩大开放，不断深化改革，转变经济增长方式，促进国民经济持续快速协调健康发展和社会全面进步。

　　今年经济社会发展的主要预期目标是:全市生产总值增长13%，其中地方增长20%；全社会固定资产投资增长22%,其中地方增长29%;社会消费品零售总额增长14%;外贸出口增长20%；吸收外商直接投资增长22%；地方财政收入增长13%；城镇居民可支配收入和农民人均纯收入分别增长8%、7%；城镇登记失业率控制在2%以内；人口自然增长率控制在7.9‰以内。实际工作中要乘势而上，加倍努力，力争发展得更快更好。

　　各位代表，做好今年的工作,我们必须以科学发展观为指导，认真落实国家宏观调控政策，善于在宏观调控中抢抓机遇，保持国民经济良好的发展势头；必须突出重点，抓住关键，在工业发展、城市建设、对外开放和农民增收等方面实现新的突破；必须大力推进科技创新，转变经济增长方式，提高区域经济质量和竞争力；必须坚持以人为本，推进统筹发展，化解社会矛盾，加快构建和谐社会；必须保护好、引导好、发挥好各方面干事创业的积极性，进一步加强油地军校结合，凝聚起强大的发展合力。只要我们全市上下进一步振奋精神，扎实苦干，我们的目标就一定能够实现，就一定能够夺取现代化建设的新胜利!

　　三、推动经济更快更好地发展

　　围绕今年的经济发展目标，重点抓好十个方面的工作。

　　(一)全面提高对外开放水平。一是提高招商工作水平。要强化专业招商，在继续调动全社会招商积极性的同时，组建行业招商队伍，加强各单位专业招商力量。强化企业招商，引导鼓励企业积极对外合资合作。强化油地校联合招商。要进一步加大招商载体建设力度，加强招商项目的筛选论证，搞好园区配套。合理确定园区配套标准和规模，降低开发成本；选准发展定位，对同行业和关联度较高的项目适当集中布局，发挥聚集效应。继续办好经贸洽谈会，丰富内容，提高实效。进一步加大项目推进落实力度，继续实行责任制，特别是对重大项目要成立强有力的推进班子，确保尽快落地建设。进一步加大投资软环境建设力度，提高办事效率和服务水平，严肃查处侵犯外来投资者和企业合法权益的行为。高度重视产业配套、人才和劳动力资源开发、融资体系建设等工作，降低综合商务成本，增强对外招商的吸引力。二是提高外贸出口水平。认真落实出口退税政策，进一步加大对出口的扶持力度，支持企业大力开拓国际市场，扩大出口规模，支持外贸企业出口本地产品，增强出口对经济增长的拉动力。实施以质取胜和市场多元化战略，优化出口商品结构，培育出口骨干企业和拳头产品，提高出口竞争力和效益。大力发展加工贸易，积极引进高水平项目，促进加工贸易升级。三是提高利用外资水平。在继续扩大利用外资规模的同时，注重提高利用外资的质量和档次，大力引进高新技术项目、产业龙头项目，引进先进技术和管理经验，嫁接改造传统产业。东营经济开发区要重点引进规模大、科技含量高的项目，提高发展的水平和带动能力。四是提高企业国际化经营水平。引导企业强化国际化经营意识，增强参与国际竞争的能力，勇于走出国门，拿资源、占市场，积极到境外承揽工程、投资办厂。鼓励支持发展劳务输出。

　　(二)加快壮大工业实力。工业是经济发展的支柱、财政增收的基础。要大力实施工业强市战略，着力培育石油化工、盐化工、精细化工、橡胶制品、纺织服装、机械电子、造纸及林木加工、食品加工八大支柱产业，加快山东加工制造业基地建设。突出抓好“四大”。一是抓大规划。搞好八大产业的发展规划，明确发展方向，引导生产要素向八大产业聚集。二是抓大企业。实施大企业带动战略，加大扶持力度，引导骨干企业深化发展战略研究，推进科技创新，培育知名品牌，加快膨胀发展，力争今年有17家企业销售收入过10亿元，其中1家过50亿元、1家过70亿元。围绕大企业积极发展配套项目，延伸产业链条，培育产业集群。三是抓大项目。引导企业围绕八大产业论证一批、建设一批、储备一批大项目，今年重点抓好50个投资过亿元的项目建设。四是抓大投入。通过政府引导和市场化运作，集中资金投向八大产业。今年地方工业固定资产投资要达到140亿元以上，其中投向八大产业的要达到125亿元以上。同时，加强对企业家的培养教育，支持企业面向国内外聘请高层次经营管理人员，努力建设一支高素质的企业家队伍。

　　(三)进一步加强“三农”工作。坚持把“三农”工作作为全部工作的重中之重，按照城乡统筹和“多予、少取、放活”的方针，落实政策，加强扶持，推动农业增效、农民增收、农村发展。一是推进农业优势产业发展壮大。鼓励农民根据市场需求，调整种养结构，发展高产高效粮食生产，发展畜牧、水产、蔬菜、冬枣、棉花等优势产业，发展规模化、集约化经营。实施优势农产品推广提高工程，大力推广应用良种和先进技术，加快建设优势农产品生产基地。继续搞好农业开发，提高农业综合生产能力，重点实施好10万亩“上农下渔”开发和20万亩土地开发整理项目。同时大力发展农村二、三产业，拓宽农民增收渠道。二是推进农业产业化、标准化、国际化。继续实行扶持政策，加快培育壮大农业龙头企业，大力发展农村合作经济组织，带动更多的农民参与产业化经营。加强农产品批发市场建设，搞活农产品流通。制定推广地方农业标准和生产技术规程，完善农产品质量监督检测网络，切实搞好黄河三角洲无规定动物疫病区建设，提高农产品质量和在国内外市场的竞争力。三是推进农村社会化服务体系建设。整合现有资源特别是乡镇一级资源，完善壮大科技、信息等服务体系，加强对农业生产的指导和服务。继续搞好千村万户农民科技培训，提高农民素质，促进农业结构调整和农村劳动力转移。四是推进农业基础设施建设和农村环境整治。继续抓好节水灌溉、防洪排涝改碱和防潮、城乡清洁用水保障、城乡污水处理“四大体系”建设，重点治理溢洪河、预备河、东营河，搞好灌区治理和田间灌排体系配套，逐步实施乡镇集中供水。基本完成300人以上村庄的规划编制，继续搞好村内道路硬化和农村沼气建设。五是推进农村各项政策的落实。认真落实农村土地承包政策，依法规范承包经营权流转。进一步落实好对种粮农民、良种和农机具购置补贴等各项政策。取消“两工”，规范一事一议筹资筹劳，减轻农民负担，并积极探索新形势下农田基本建设的新机制、新办法。加强农村集体资产经营管理，在有条件的村推行规范化、公司化管理。

　　(四)积极发展服务业。继续搞好服务业设施建设，完善商品流通体系，推广连锁经营、电子商务等现代流通方式，提高商贸流通业的档次和水平。搞好规划，积极发展现代物流业。围绕实施黄河口生态旅游、滨海旅游、油城旅游、文化旅游四大工程，抓好黄河口湿地生态园、生态农业观光园、黄河河口物理模型等项目的规划建设，整合旅游资源，加强宣传促销，完善旅游服务体系，加快发展旅游业。大力发展中介服务、社区服务、文化娱乐等产业。抓住服务业领域对外开放的机遇，引进服务项目。积极发展消费信贷，引导居民扩大消费。

　　(五)大力发展民营经济。认真落实扶持民营经济的政策措施，进一步加强宣传发动，引导更多的人搞民营、办实业，壮大创业群体。放宽市场准入，拓宽民营经济发展空间。引导现有民营企业不断增投入、上项目、扩规模，积极推进机制、科技、管理创新，参与国际竞争，加快发展壮大。坚持政府引导、社会参与、规范服务、市场运作的原则，加快建立融资担保、信用评价、人才培训、技术信息、法律服务等服务体系，积极发展行业协会。坚持民营企业与其他企业一视同仁，坚决治理“三乱”，切实保护民营企业的合法权益。

　　(六)加强城市和基础设施建设。深化城市发展战略研究，抓住建设山东半岛城市群的机遇，推动城市规划、建设、管理、经营全面上水平，努力把东营建设成为最适宜创业发展、最适宜人类居住的城市。一是提高规划设计水平。今年完成城市总体规划修编，完成中心城所有专业专项规划编制，深化细化详规，高水平地搞好标志性地段、标志性建筑的设计。二是提高城市建设水平。围绕完善城市功能、丰富文化内涵、提高城市品位、增强辐射带动能力，集中力量、集中资金建设一批重点工程；继续搞好城区道路建设改造，配套完善市政公用设施，加快东西城对接区建设和城中村改造；进一步搞好绿化美化亮化，改造完善城市水系，综合整治广利河、东营河、五六干合排等河道城区段。积极推行物业管理。继续搞好创建全国无障碍设施示范城工作。三是提高城市管理水平。完善管理体系，创新管理方式，建立管理长效机制。加大执法管理力度，搞好城市交通秩序、市容市貌、环境卫生、市场秩序等综合整治，改善城市环境。四是提高经营城市水平。盘活城市存量资产，吸引社会投资，推动城市发展。进一步加强县城和小城镇建设，推进城市化进程。

　　加强基础设施建设。围绕构建交通大通道，加快推进“两港”、“两路”建设。“两港”就是加快实施东营港2&times;3万吨码头扩建，搞好东营永安机场建设并争取增加航线航班；“两路”就是推进环渤海高速公路和黄大铁路建设。同时，搞好干线公路和农村公路建设改造。做好大唐东营发电厂一期工程、鲁能东营风力发电项目和东营500千伏输变电工程的前期工作。继续搞好“数字化东营”建设。

　　(七)切实做好财税金融和审计工作。大力培植财源，扶持发展有利于增加地方财政收入的优势产业和项目。强化税收征管，切实做到应收尽收，把经济发展成果体现到财政收入上来。加强各种规费的征收管理，确保该减的减到位，该收的收到位。调整优化支出结构，集中财力办大事、保重点。继续加大对“三农”、社会保障、重点社会事业等的倾斜力度，逐步建立规范的转移支付制度。今年市财政安排支持基层和“三农”的资金4.9亿元。牢固树立过紧日子的思想，坚决反对大手大脚、铺张浪费。高度重视解决部分县乡财政困难问题，认真落实促强扶弱的政策措施，大力发展县域经济，壮大县乡财政实力，并积极稳妥地调整完善县乡财政体制。进一步深化预算改革，强化预算约束，完善财政集中支付、政府采购等制度，严格执行收支两条线管理规定。强化审计监督，加强预算执行审计、经济责任审计、投资效益审计和专项审计，坚决查处违规违纪问题。积极支持金融机构的改革和发展，壮大金融体系，改善金融服务，密切银企关系，为经济发展提供支持。要加快发展必须不断地加大投入。要积极拓宽融资渠道，建立多元投入机制，确保投入的增长与发展目标相适应。

　　(八)不断深化各项改革。进一步深化企业改革，积极推行经营管理集团控股、经营者持大股、职工自愿参股的股份制改造，优化股权结构，完善法人治理结构，健全激励约束机制，加快建立现代企业制度。积极推进企业上市，大力培植上市后备资源。深化国有资产管理体制改革，依法加强对国有资产的监管。认真落实投资体制改革的政策措施，保证企业的投资自主权，加强政府投资管理。切实用好开发银行贷款。进一步深化农村税费改革和粮食流通体制改革。搞好价格等方面的改革。加快资本、劳动力、技术等要素市场和社会信用体系建设，培育规范市场中介组织，完善现代市场体系。积极稳妥地推进事业单位综合配套改革和市政公用事业改革。继续整顿和规范市场经济秩序，加大市场监管力度，搞好专项整治。深入实施食品药品放心工程。

　　(九)努力推动科技进步。完善科技推广服务体系，加快高新技术和先进实用技术在各行各业的应用，推进工业信息化，带动产业结构优化升级。加强区域科技创新体系建设，引导扶持企业建设和完善技术开发中心，提高技术创新能力。进一步拓展产学研结合领域，搞好科技成果的引进转化，大力发展高新技术产业。科技发展关键在人才。要牢固树立人才资源是第一资源的观念，切实加强行政人才、企业经营管理人才和专业技术人才队伍建设，加大实用技能人才、农村技术人才的培养力度，加强劳动力资源开发，全面提高劳动者素质。创新人才工作机制，努力为各类人才施展才能创造条件。

　　(十)推进区域经济社会一体化。高举团结发展的旗帜，进一步深化油地军校结合。大力推进区域经济社会一体化，积极支持油田“油气当量重上三千万”、提升经济运行质量、提高职工生活水平，支持石油大学和济军生产基地加快改革和发展，支持企业间合资合作，优势互补，共同发展。认真落实支持油田存续企业改制的政策措施，搞好油田工业园建设，促进油田存续企业发展壮大。进一步做好油田帮扶乡镇工作。积极稳妥地做好油田中小学移交工作。大力推进城市规划建设管理一体化，共同搞好重点工程建设。大力推进基础设施建设一体化，统筹规划建设一批基础设施项目。现在，油地军校结合的形势越来越好，我们要倍加珍惜，倍加维护，倍加努力，共同推动繁荣发展，共同建设幸福家园，共同创造东营的美好未来!

　　四、努力构建社会主义和谐社会

　　构建和谐社会是人们的美好追求，是全面建设小康社会的必然要求，是实现长治久安的治本之策。在加快发展的进程中，我们必须坚持以人为本，努力建设健康、公平、和谐、稳定的社会。

　　(一)实现好维护好发展好广大群众的根本利益，促进社会公平公正。坚持把群众的根本利益作为制定政策措施、开展工作的出发点和落脚点，正确反映和兼顾不同方面群众的利益，让所有社会成员共享改革和发展的成果。切实做好就业再就业工作，认真落实优惠政策，千方百计增加就业岗位，通过充分就业普遍增加群众的收入。规范完善用工制度，所有用工都要按照规定签订劳动合同，参加社会保险，落实劳动保护和安全防护措施。严格执行最低工资标准，建立工资按时发放的监督保障机制，严禁拖欠克扣职工工资。完善社会保障救助体系，重点抓好“五保五救助”。“五保”即：扩大城乡养老保险覆盖面，建立失地农民养老保险制度，逐步形成覆盖城乡所有劳动者的养老保险机制；完善农民、城镇职工医疗保险及大病救助制度，建立城镇其他居民医疗保险及大病救助制度，逐步实现医疗保险的全面覆盖；逐步提高城乡低保、失业保险和农村“五保”老人集中供养水平。“五救助”即：完善教育救助制度，继续对新考入大学的贫困学生实施救助，免除城乡低保户子女就学的各种费用，免费为盲、聋哑、弱智儿童提供上学机会，视情减免其他贫困学生的上学费用；建立城乡老年人救助制度；继续搞好残疾人救助、灾害救助、城乡居民住房救助。加强扶贫工作，加快贫困群众脱贫致富步伐。切实保护广大群众的合法权益，坚决纠正和防止征地、拆迁、改制中损害群众利益的行为。

　　(二)加强精神文明和民主法制建设，促进社会全面进步。加强思想道德建设，在全社会大力弘扬“和谐、诚信、创新、卓越”的东营精神，营造平等友爱、融洽和谐的人际环境。广泛开展群众性精神文明创建活动，深入推进创建“文明城市”、“小康文明村”工作和“信用东营”建设。加强民主法制建设。各级政府要自觉接受人大及其常委会的监督，认真落实人大及其常委会的决议决定，支持人民政协履行政治协商、民主监督、参政议政职能，认真办理人大代表建议和政协提案，加强与各民主党派、工商联、人民团体和社会各界的联系。扩大基层民主，完善村(居)民自治制度，保证群众依法行使民主权利。大力推进依法治市，深入开展普法教育，提高全民的法律素质。全面发展各项社会事业。进一步落实教育优先发展的战略地位，提高义务教育实施水平，积极推行学前三年教育，加快普及普通高中教育，大力发展职业教育和成人教育。完善疾病预防控制体系和医疗救治体系，建设公共卫生信息体系和卫生监督执法体系，提高卫生服务水平和突发公共卫生事件应急处理能力。加快文化设施建设，充分挖掘文化资源，繁荣文化事业，大力发展文化产业。广泛开展全民健身运动，提高全民健康水平和竞技体育水平。全面发展广播电视、新闻出版事业。关心支持妇女儿童、残疾人事业，做好老龄工作。加强全民国防教育，抓好民兵预备役建设，巩固扩大双拥共建成果。进一步做好外事侨务、民族宗教、无线电管理、档案、气象、对台、地名、史志、人防、防震和减灾救灾等工作。

　　(三)做好人口、资源、环境工作，促进人与自然和谐发展。坚持不懈地抓好人口和计划生育工作，稳定低生育水平，促进优生优育，提高人口素质。依法加强土地管理，严格保护耕地。广泛开展资源节约活动，提高资源利用效益，建设节约型社会。认真实施生态市建设规划，加强环保基础设施建设，加大老污染源治理力度，严格控制新污染源产生。大力发展循环经济，推行清洁生产。大搞植树造林，实施好千村绿化及公路、水系绿化等工程，加快建设“绿色东营”。搞好土地次生盐碱化治理，改善生态条件。加强自然保护区和地质公园建设管理，搞好湿地生态和地质遗迹保护。深入开展创建生态县区和环境优美乡镇、村活动，改善城乡生态面貌。

　　(四)推进“平安东营”建设，促进社会稳定。坚持稳定压倒一切，打防并举，标本兼治，建立完善维护稳定的长效机制。强化基层基础工作，着重完善矛盾纠纷排查调处机制，努力做到各种矛盾纠纷发现得早、化解得了、控制得住、处置得好。高度重视做好信访工作，积极预防和妥善处置群体性事件，对群众反映的问题，落实措施，落实责任，及时加以解决。大力加强社会治安综合治理，深入开展“严打”整治斗争，集中侦破大案要案，依法严厉打击各种犯罪行为。严厉打击各类“霸头”恶势力，搞好施工环境整治。认真落实安全生产责任制，加强安全教育，加大安全投入，强化监督检查，搞好重点领域的专项整治，坚决遏制重特大事故的发生。建立完善社会灾害和自然灾害的预警、应急机制，提高应对突发事件和风险的能力。

　　各位代表，在全面推进各项工作的同时，我们将继续为民办好十件实事：一是完善“五保五救助”社会保障救助体系；二是继续建设“平安东营”；三是继续搞好荒碱地开发及土地整理；四是全面实施农民增收工程；五是全面取消农业税，让全市百万农民彻底摆脱负担了几千年的“皇粮国税”；六是全面实施农村中小学标准化建设；七是继续推进“小康文明村”建设；八是实施水利三排及涝洼地治理；九是实施乡镇集中供水示范工程，提高20万农民的饮水质量；十是建设电子综合服务应用平台和便民服务设施。同时，根据全市经济社会发展的需要，建设十大工程：一是建设改造城乡道路；二是建设环渤海高速公路，争取开工建设黄大铁路；三是扩建东营港；四是继续搞好开发区基础设施配套建设；五是建设观海栈桥；六是建设文化艺术中心；七是建设会展中心；八是建设东营中心渔港；九是改造治理中心城水系及城区河道；十是继续搞好黄河南海堤建设。这十件实事和十大工程，投资数额巨大，对东营的当前和长远发展影响深远，我们将严格实行领导、承办单位、项目法人责任制，严格实行招标投标制，严格实行工程监理制，强化督查调度，确保说到做到，按期完成，取信于民!

　　各位代表，编制“十一五”规划和2020年远景目标，是今年的一项重要工作。我们将组织力量，深入研究论证，编制好规划，勾画好今后一个时期的发展蓝图，以此凝聚和鼓舞全市人民向着现代化建设的宏伟目标奋勇前进。

　　五、切实加强政府自身建设

　　面对新形势、新任务,我们必须进一步加强政府自身建设,深化行政管理体制改革，加快转变政府职能和行政管理方式，全面提高行政能力,努力建设责任政府、阳光政府、服务政府、法治政府。

　　大力提高依法行政水平。各级政府都要增强宪法和法律意识，自觉维护宪法和法律的尊严，真正把依法行政作为工作的基本准则，贯穿于行政管理的各个环节。要严格按照法定权限和程序行使权力，切实履行好经济调节、市场监管、社会管理、公共服务职能，善于运用法律手段管理经济和社会事务。进一步加强和改善行政执法工作，转变执法方式，规范执法行为，严格执法、公正执法、文明执法，把依法加强监督管理与热情服务有机统一起来。强化执法监督，积极推行行政执法责任制和执法过错责任追究制，建立公开公平公正的评议考核制，切实做到有权必有责、用权受监督、侵权须赔偿、违法要追究。按照公开、便民、高效的要求，深化行政审批制度改革，抓好行政审批服务大厅建设，全面推行政务公开、服务承诺等制度，规范工作程序，加快发展电子政务，降低行政成本，提高行政效率和服务水平。

　　健全完善决策目标、执行责任、考核监督“三个体系”。要完善群众参与、专家咨询和政府决策相结合的行政决策机制，明确决策权限，遵守决策程序，坚持重大问题集体决策制度、专家咨询论证制度、社会公示和听证制度，建立健全决策责任追究制度，确保依法决策、科学决策、民主决策。完善主体清晰、责任明确、权责统一的执行责任体系，将经济社会发展的各项目标任务细化量化，逐项落实到人，实行严格的责任制，确保执行有力。改进考核评价体系，完善政务督查考核、行政效能监察、社会评议部门等制度，强化监督考核手段，确保各项工作落到实处。

　　努力建设勤政为民的公务员队伍。教育各级公务员牢固树立勤政为民的理念，想问题、作决策、办事情都要始终把群众的利益放在首位，认真倾听群众呼声，积极解决群众关注的热点难点问题，真正做到权为民所用、情为民所系、利为民所谋。加强学习培训工作，促使各级公务员不断掌握新知识，积累新经验，增长新本领，努力提高为人民服务的能力和水平。完善考核奖惩和竞争机制，激励广大公务员解放思想、干事创业，争创一流业绩。切实改进工作作风，大兴求真务实之风，精简会议和文件，减少应酬活动，深入基层，加强调查研究，解决实际问题，提高工作实效，坚决防止和克服官僚主义、形式主义，反对弄虚作假，努力使每一项工作都经得起实践的检验、群众的检验和历史的检验。坚持从严治政，认真落实廉政建设责任制,进一步抓好领导干部廉洁自律,坚决查处违纪违法案件,坚决纠正损害群众利益的不正之风，坚决惩处腐败分子。建立健全教育、制度、监督并重的惩治和预防腐败体系,加大从源头上预防和治理腐败的力度。各级公务员特别是领导干部要加强世界观改造,牢记“两个务必”，把廉洁作为立身之本、为政之德、行为之范,严格执行“四大纪律、八项要求”、“五个不许”和廉洁从政的各项规定，廉洁奉公，勤政为民。

　　各位代表!我们肩负的任务光荣而艰巨，责任神圣而重大。让我们紧密团结在以胡锦涛同志为总书记的党中央周围,高举邓小平理论和“三个代表”重要思想伟大旗帜,在中共东营市委的坚强领导下,进一步解放思想,振奋精神,凝心聚力,干事创业,为实现全面建设小康社会和现代化建设的宏伟目标而努力奋斗!
